# Antitrypanosomal Activity of 1,2,3-Triazole-Based Hybrids Evaluated Using In Vitro Preclinical Translational Models

**DOI:** 10.3390/biology12091222

**Published:** 2023-09-08

**Authors:** Lorraine Martins Rocha Orlando, Leonardo da Silva Lara, Guilherme Curty Lechuga, Giseli Capaci Rodrigues, Omar Ginoble Pandoli, Druval Santos de Sá, Mirian Claudia de Souza Pereira

**Affiliations:** 1 Laboratório de Ultraestrutura Celular, Instituto Oswaldo Cruz, Fiocruz Av. Brasil 4365, Rio de Janeiro 21040-900, Brazil; lorrainemartins07@hotmail.com (L.M.R.O.); leonardosilva.lara@hotmail.com (L.d.S.L.); guilherme.lechuga@yahoo.com.br (G.C.L.); 2 Programa de Pós-Graduação em Ensino das Ciências, Unigranrio Rua Prof. José de Souza Herdy, Duque de Caxias, Rio de Janeiro 25071-970, Brazil; giselicapaci@gmail.com; 3 Departamento de Química, Pontifícia Universidade Católica, Rua Marquês de São Vincente, 225, Rio de Janeiro 22451-900, Brazil; omarpandoli@puc-rio.br (O.G.P.); drdruval@gmail.com (D.S.d.S.); 4 Dipartimento di Farmacia, Università degli Studi di Genova, Viale Cembrano 4, 16126 Genova, Italy

**Keywords:** chagas disease, *Trypanosoma cruzi*, triazole, drug candidates

## Abstract

**Simple Summary:**

Chagas disease, caused by the protozoan *Trypanosoma cruzi*, is a neglected tropical disease that affects 6–7 million people worldwide. It is a global disease, due to migration from Latin America to other regions of the world, and a recognized worldwide public health problem. Clinical treatment is based on two fifty-year-old drugs, nifurtimox and benznidazole. These drugs have low efficacy in the chronic phase of the disease and have severe adverse effects, making the search for new drugs essential. This study aimed to evaluate the trypanocidal potential of 1,2,3-triazole analogs. Our data highlight three analogs with potent activity against trypomastigotes and similar efficacy to benznidazole, the reference drug, against intracellular parasites. These analogs showed high efficacy in 3D cardiac microtissue. However, despite potentially reducing parasite load, the promising candidates did not inhibit the resurgence of the parasite in the absence of the drug. Newly designed analogs will be screened against *T. cruzi* to identify potentially active and safe drugs for Chagas disease therapy.

**Abstract:**

Chagas disease therapy still relies on two nitroderivatives, nifurtimox and benznidazole (Bz), which have important limitations and serious adverse effects. New therapeutic alternatives for this silent disease, which has become a worldwide public health problem, are essential for its control and elimination. In this study, 1,2,3-triazole analogues were evaluated for efficacy against *T. cruzi*. Three triazole derivatives, **1d** (0.21 µM), **1f** (1.23 µM), and **1g** (2.28 µM), showed potent activity against trypomastigotes, reaching IC_50_ values 10 to 100 times greater than Bz (22.79 µM). Promising candidates are active against intracellular amastigotes (IC_50_ ≤ 6.20 µM). Treatment of 3D cardiac spheroids, a translational in vitro model, significantly reduced parasite load, indicating good drug diffusion and efficacy. Oral bioavailability was predicted for triazole derivatives. Although infection was significantly reduced without drug pressure in a washout assay, the triazole derivatives did not inhibit parasite resurgence. An isobologram analysis revealed an additive interaction when 1,2,3-triazole analogs and Bz were combined in vitro. These data indicate a strengthened potential of the triazole scaffold and encourage optimization based on an analysis of the structure–activity relationship aimed at identifying new compounds potentially active against *T. cruzi*.

## 1. Introduction

Neglected tropical diseases (NTDs), a group of 20 diseases recognized by the World Health Organization (WHO), impact over 1 billion people in the world, mainly the poorer population living with inadequate sanitation and poor social and economic conditions and without access to adequate healthcare [1]. For decades, NTDs have been overlooked by the pharmaceutical industry due to a lack of effective and safe drug treatment, stemming from the high cost of drug development and an inherent risk of failure. Despite the success in controlling and eradicating some NTDs in the period 2011–2020, such as Guinea worm infection, which was eradicated from 42 countries, new prospective proposals have been launched by the WHO for the period 2021–2030 [2]. In this challenging scenario, Chagas disease deserves renewed attention.

This infectious, potentially fatal disease, caused by the protozoan *Trypanosoma cruzi*, is the main cause of cardiomyopathy in endemic countries in Latin America and is responsible for 41% of heart failures in endemic areas [3]. Usually asymptomatic and without classic signs in the acute phase, it is difficult to diagnose and often goes unnoticed and progresses to the chronic phase. Infected individuals may remain asymptomatic (indeterminate form) or evolve after decades into the symptomatic form with cardiac, gastrointestinal (megacolon and megaesophagus), neurological, or cardio-digestive manifestations [4,5]. Chronic Chagas cardiomyopathy (CCC), the main clinical manifestation, affects 20–30% of infected individuals, manifesting as low-intensity myocarditis, fibrosis, and cardiac conduction system damage, potentially leading to heart failure (HF) and sudden death [6]. Based on the level of ventricular dysfunction and degree of HF in the chronic phase, cardiovascular involvement is classified into five stages: A, B1, B2, C, and D. Individuals with no cardiac alteration are classified as stage A. Stage B1 individuals are asymptomatic but present electrocardiographic (ECG) alteration. Stage B2 individuals are asymptomatic with left ventricular dysfunction and no HF. Stage C patients have left ventricular dysfunction and HF. Stage D patients have symptoms of HF at rest, refractory to maximized medical therapy [7]. Sudden death is highlighted as a conspicuous feature (60%) in carriers of Chagas disease [8]. It is noteworthy that antiarrhythmic drugs effectively reduce ventricular arrhythmias but are not successful in reducing mortality [9,10]. Prevention of transmission, expansion of diagnostic testing, and access to treatment and clinical care remain important for the management of the disease [11].

Effective new drugs for the treatment of Chagas disease are yet to be discovered, however. Benznidazole (Bz) and nifurtimox (NFX), introduced more than 50 years ago, are the only drugs currently used in clinical therapy. They have significant drawbacks, including low tolerability, with adverse effects that often lead to discontinuation of treatment [4], partial effectiveness in the acute phase (60–80%), probably due to the variable drug susceptibility among *T. cruzi* strains, and low efficacy in the chronic phase of the disease [12]. In addition, cardiomyopathy progression was not prevented in patients with chronic Chagas cardiomyopathy treated with Bz [13]. The latest clinical trials revealed the therapeutic failure of potential treatment candidates. Posaconazole and fosravuconazole (a prodrug of ravuconazole) did not sustain parasite clearance in monotherapy [14,15], and although the combination with Bz resulted in an effective antiparasitic response, it did not improve Bz monotherapy efficacy [16,17]. Reduced dose and shorter course regimens in a 2-week Bz treatment showed similar efficacy to the Bz standard regimen but with lower serious side effects and treatment discontinuation, becoming a promising treatment regimen for Chagas disease [17]. The recently released results from the fexinidazole clinical trial, an FDA-approved drug for the treatment of human African trypanosomiasis [18], revealed a rapid parasitological clearance but accompanied by serious adverse effects [19]. Thus, safe and more efficacious drugs to combat Chagas disease are still urgently needed.

Drug repurposing, new drug development, and optimization have been proposed to identify potent candidate drugs against *T. cruzi* with prospects for advancing in preclinical and clinical trials. Triazoles, a class of five-member nitrogen heterocyclic compounds with broad and potent bioactivity, have been highlighted for their antifungal, antiviral, antioxidant, anti-inflammatory, antitumor, and antiparasitic properties [20]. Triazole activity has been reported in infectious diseases, including Chagas disease, malaria, tuberculosis, and leishmaniasis [21]. Multiple mechanisms of action have been reported based on triazole activity against *T. cruzi*, including cruzain, sterol 14α demethylase (CYP51), and trans-sialidase inhibitors [21]. Nitrotriazole derivatives exhibit potent bioactivity against many microorganisms, especially trypanosomatid parasites [22]. Analogs of 1,2,3-triazole-2-nitroimidazole also demonstrated anti-*T. cruzi* activity, showing high potency and selectivity [23]. In this study, *in silico* approaches and in vitro preclinical assays with more predictive models of translational efficacy were used to evaluate the activity of 1,2,3-triazole analogs against *T. cruzi*. Three active 1,2,3-triazole derivatives stood out in the data for prospective hit-to-lead optimization.

## 2. Materials and Methods

### 2.1. Chemistry

**Scheme 1:**
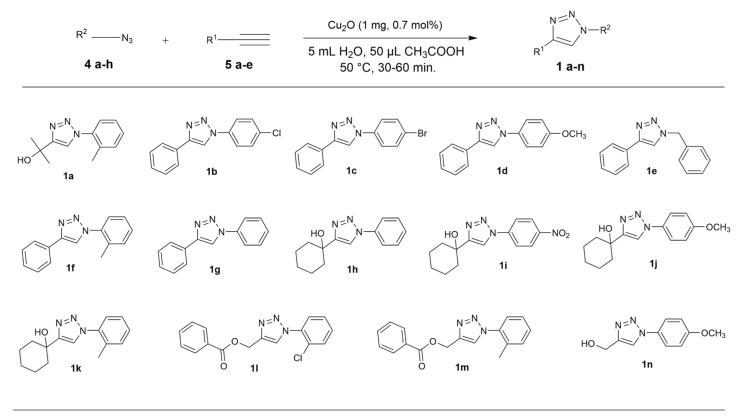
Condition of the CuAAC reaction between aryl azides (**4a**–**h**) and alkynes (**5a**–**e**) to obtain 1,4-disubstituted 1,2,3-triazole (**1a**–**n**).

The 1,4-disubstituted 1,2,3-triazoles (**1a**–**n**) structures were designed to investigate the impact of electron-donating and electron-withdrawing functional groups on the triazole ring and, consequently, their anti-*T. cruzi* activity. For this purpose, aryl azides ortho-substituted with -CH_3_ and -Cl, as well as para-substituted with -CH_3_, -OCH_3_, -Br, and NO_2_, along with aromatic and aliphatic alkynes containing an -OH group, were used. The synthesis of all 1,2,3-triazoles was performed using the adjusted methodology of Shao and co-workers [24]. In Scheme 1, 1.0 mmol of arylazide (**4a**–**h**), 1.1 mmol of alkyne (**5a**–**e**), and 5 mL of H_2_O were stirred with 50 µL of glacial acetic acid (HAC) for 60 s. Then, 1 mg of Cu_2_O (0.7 mol%) was added to the mixture, which was stirred at room temperature for 30–60 min. Subsequently, the mixture was extracted with ethyl acetate (3 × 15 mL) and then the combined organic phases were dried with Na_2_SO_4_ and concentrated under reduced pressure to obtain the corresponding 1,4-disubstituted 1,2,3-triazole. The triazoles **1d**, **1h**, **1i**, **1j**, and **1k** were crystallized from a mixture of acetonitrile and hexane (1:4). The purity of the **1a**–**n** triazoles was determined with absolute quantification using ^1^H NMR with methyl methanesulfonate as an internal standard [25]. The detailed methodology and calculations used to determine the purity of the compounds are provided in the Supplementary Material. Melting points (mp) were determined using analog model equipment from Fisatom (São Paulo, Brazil). Fourier transform infrared (FT-IR) spectra were acquired using a Bruker ALPHA II spectrometer (Bruker, Rheinstetten, Germany) in the wavenumber range of 400 to 4000 cm^−1^, with a spectral resolution of 4 cm^−1^. Nuclear magnetic resonance (NMR) spectra were obtained using a Bruker Avance instrument (Bruker, Rheinstetten, Germany) operating at 400 MHz for ^1^H NMR and 100 MHz for ^13^C NMR at a temperature of 25 °C. High-resolution mass spectrometry (HRMS) spectra were recorded using a Micromass/Waters ZQ-4000 spectrometer (Waters, Milford, MA, USA) (Supplementary Figure S1).

### 2.2. General Procedure for CuAAC of Organic Azides with Alkynes to 1,4-Disubstituted 1,2,3-Triazoles *(**1a**–**n**)*

Characterization of 2-(1-(*o*-Tolyl)-1*H*-1,2,3-triazole-4-yl)propan-2-ol (**1a**) [26]:

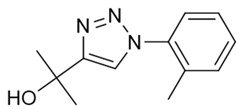



Triazole **1a** was isolated as a pale brown solid (yield 90%, purity: 94.0%). mp: 120–122 °C; FT-IR υ (cm^−1^): 3336, 3120, 2974, 2927, 1582, 1498; **^1^H NMR** (400 MHz, DMSO-d6) δ 8.20 (s, 1H), 7.47 (dd, *J* = 4.4, 2.0 Hz, 2H), 7.40 (dd, *J* = 4.3, 2.0 Hz, 2H), 5.21 (s, 1H), 2.16 (s, 3H), 1.54 (s, 6H); **^13^C NMR** δ 156.28, 136.97, 133.43, 131.78, 130.01, 127.39, 126.38, 122.60, 67.46, 31.13, 17.94; HRMS (ESI): *m/z* [M+Na] calcd. for C_12_H_15_N_3_NaO: 240.110733, found: 240.110674.

Characterization of 1-(4-Chlorophenyl)-4-phenyl-1*H*-1,2,3-triazole (**1b**) [26]:

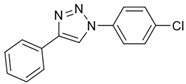



Triazole **1b** was isolated as a pale-yellow solid (yield 90%, purity: 97.0%). mp: 218–220 °C; FT-IR υ (cm^−1^): 3120, 3095 3052, 1500, 1480; **^1^H NMR** (400 MHz, DMSO-d6) δ 9.33 (s, 1H), 8.03–7.98 (m, 2H), 7.97–7.91 (m, 2H), 7.75–7.69 (m, 2H), 7.55–7.48 (m, 2H), 7.43–7.37 (m, 1H); **^13^C NMR** (101 MHz, DMSO) δ 147.92, 135.90, 133.45, 130.54, 130.40, 129.51, 128.83, 125.82, 122.15, 120.18; HRMS (ESI): *m*/*z* [M+Na] calcd. for C_14_H_10_ClN_3_Na: 278.045546, found: 278.044930.

Characterization of 1-(4-Bromophenyl)-4-phenyl-1*H*-1,2,3-triazole (**1c**) [26]:

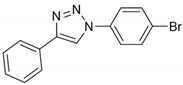



Triazole **1c** was isolated as a white solid (yield 90%, purity: 98.5%). mp: 222–225 °C; FT-IR υ (cm^−1^): 3120, 3095 3056, 2960, 2917, 1500, 1480; **^1^H NMR** (400 MHz, DMSO-d6) δ 9.33 (s, 1H), 7.94 (dd, *J* = 8.6, 1.7 Hz, 4H), 7.88–7.82 (m, 2H), 7.51 (dd, *J* = 8.4, 7.0 Hz, 2H), 7.43–7.37 (m, 1H); **^13^C NMR** (101 MHz, DMSO) δ 147.94, 136.29, 133.32, 130.52, 129.51, 128.84, 125.82, 122.38, 121.82, 120.12; HRMS (ESI): *m*/*z* [M+Na] calcd. for C_14_H_10_BrN_3_Na: 321.995030, found: 321.994100.

Characterization of 1-(4-Methoxyphenyl)-4-phenyl-1*H*-1,2,3-triazole (**1d**) [26,27]:

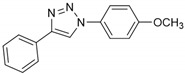



Triazole **1d** was isolated as a white solid (yield 92%, purity: 99.8%). mp: 159–160 °C. FT-IR υ (cm^−1^): 3120, 3095 2960, 2935, 2837, 1607, 1515; **^1^H NMR** (400 MHz, DMSO-d6) δ 9.19 (s, 1H), 7.97–7.92 (m, 2H), 7.89–7.83 (m, 2H), 7.53–7.47 (m, 2H), 7.38 (t, *J* = 7.4 Hz, 1H), 7.21–7.15 (m, 2H), 3.85 (s, 3H); **^13^C NMR** (101 MHz, DMSO) δ 159.14, 146.94, 130.19, 129.89, 128.81, 127.99, 125.12, 121.52, 119.44, 114.76, 55.43; HRMS (ESI): *m*/*z* [M+Na] calcd. for C_15_H_13_N_3_NaO: 274.095083, found: 274.094521.

Characterization of 1-Benzyl-4-phenyl-1*H*-1,2,3-triazole (**1e**) [28]:

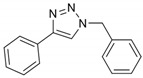



Triazole **1e** was isolated as a white solid (yield 95%, purity: 99.5%). mp: 123–124 °C. FT-IR υ (cm^−1^): 3032, 2931, 2921, 2853, 1603, 1455, 1334; **^1^H NMR** (400 MHz, DMSO-d6) δ 8.64 (s, 1H), 7.88–7.82 (m, 2H), 7.48–7.30 (m, 8H), 5.65 (s, 2H); **^13^C NMR** (101 MHz, DMSO) δ 147.12, 136.47, 131.10, 129.36, 129.27, 128.64, 128.37, 128.35, 125.62, 122.03, 53.50; HRMS (ESI): *m*/*z* [M+Na] calcd. for C_15_H_13_N_3_Na: 258.100168, found: 258.100341.

Characterization of 4-Phenyl-1-(m-tolyl)-1*H*-1,2,3-triazole (**1f**):

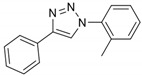



Triazole **1f** was isolated as an orange solid (yield 79%, purity: 99.7%). mp: 83–84 °C. FT-IR υ (cm^−1^): 3122, 3095 3052, 3021, 2917, 2849, 1607, 1591; **^1^H NMR** (400 MHz, DMSO-d6) δ 9.28 (s, 1H), 7.99–7.93 (m, 2H), 7.80 (s, 1H), 7.74 (s, 1H), 7.51 (td, *J* = 7.8, 3.0 Hz, 3H), 7.42–7.31 (m, 2H), 2.45 (s, 3H); **^13^C NMR** (101 MHz, DMSO) δ 147.71, 140.16, 137.06, 130.73, 130.21, 129.78, 129.47, 128.70, 125.79, 120.87, 120.03, 117.57, 21.42; HRMS (ESI): *m*/*z* [M+Na] calcd. for C_15_H_13_N_3_Na: 258.100168, found: 258.100587.

Characterization of 1-(4-Diphenyl)-1*H*-1,2,3-triazole (**1g**) [26,29]:

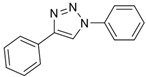



Triazole **1g** was isolated as an off-white solid (yield 89%, purity: 99.6%). mp: 171–172 °C; FT-IR υ (cm-1): 3119, 3084 3052, 2945, 2909, 1498, 1475; **^1^H NMR** (400 MHz, DMSO-d6) δ 9.31 (s, 1H), 8.00–7.93 (m, 4H), 7.65 (dd, *J* = 8.6, 7.2 Hz, 2H), 7.57–7.48 (m, 3H), 7.43–7.37 (m, 1H); **^13^C NMR** (101 MHz, DMSO) δ 147.79, 137.10, 130.70, 130.42, 129.48, 129.21, 128.73, 125.82, 120.48, 120.09; HRMS (ESI): *m*/*z* [M+Na] calcd. for C_14_H_11_N_3_Na: 244.084518, found: 244.085003.

Characterization of 1-(1-Phenyl-1*H*-1,2,3-triazole-4-yl)cyclohexan-1-ol (**1h**) [30]:

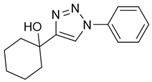



Triazole **1h** was isolated as a light white solid (yield 89%, purity: 99.8%). mp:188–190 °C; FT-IR υ (cm^−1^): 3223, 3101 3056, 2927, 2906, 2851, 1597, 1498; **^1^H NMR** (400 MHz, DMSO-d6) δ 8.62–8.57 (m, 1H), 7.95–7.87 (m, 2H), 7.59 (t, *J* = 7.9 Hz, 2H), 7.51–7.45 (m, 1H), 5.02 (s, 1H), 2.02–1.90 (m, 2H), 1.83–1.72 (m, 3H), 1.68 (d, *J* = 10.5 Hz, 1H), 1.47 (s, 1H), 1.38–1.28 (m, 1H); **^13^C NMR** (101 MHz, DMSO) δ 157.24, 137.29, 130.32, 128.80, 120.28, 119.73, 68.43, 38.13, 25.70, 22.16. HRMS (ESI): *m*/*z* [M+Na] calcd. for C_14_H_17_N_3_NaO: 266.126383, found: 266.127590.

Characterization of 1-(1-(4-Nitrophenyl)-1*H*-1,2,3-triazole-4-yl)cyclohexan-1-ol (**1i**) [30]:

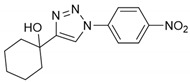



Triazole **1i** was isolated as a light white solid (yield 89%, purity 99.7%). mp: 209–210 °C. FT-IR υ (cm^−1^): 3216, 3116, 3067, 2935, 2853, 1597, 1525, 1504; **^1^H NMR** (400 MHz, DMSO-d6) δ 8.84 (s, 1H), 8.48–8.40 (m, 2H), 8.29–8.22 (m, 2H), 5.11 (s, 1H), 2.03–1.91 (m, 2H), 1.74 (dt, *J* = 33.7, 11.8 Hz, 4H), 1.60–1.40 (m, 3H), 1.32 (q, *J* = 10.2 Hz, 1H); **^13^C NMR** (101 MHz, DMSO) δ 156.79, 145.86, 140.47, 124.97, 119.69, 119.22, 67.37, 36.96, 24.59, 21.05; HRMS (ESI): *m*/*z* [M+Na] calcd. for C_14_H_16_N_4_NaO_3_: 311.111461, found: 311.111942.

Characterization of 1-(1-(4-Methoxyphenyl)-1*H*-1,2,3-triazole-4-yl)cyclohexan-1-ol (**1j**):

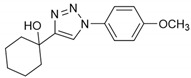



Triazole **1j** was isolated as a pale-yellow solid (yield 89%, purity: 99.8%). mp: 174–175 °C; FT-IR υ (cm^−1^): 3221, 3101, 3056, 2929, 2851, 2833, 1611, 1591, 1541, 1515; **^1^H NMR** (400 MHz, DMSO-d6) δ 8.48 (s, 1H), 7.80 (d, *J* = 9.1 Hz, 2H), 7.12 (d, *J* = 9.0 Hz, 2H), 4.99 (s, 1H), 3.83 (s, 3H), 1.95 (t, *J* = 11.9 Hz, 2H), 1.74 (dd, *J* = 28.3, 15.1 Hz, 4H), 1.54 (s, 1H), 1.49–1.41 (m, 2H), 1.38–1.27 (m, 1H); **^13^C NMR** (101 MHz, DMSO) δ 159.46, 157.01, 130.76, 121.93, 119.69, 115.29, 68.44, 55.99, 38.15, 25.70, 22.17; HRMS (ESI): *m*/*z* [M+Na] calcd. for C_15_H_19_N_3_NaO_2_: 296.136948, found: 296.137228.

Characterization of 1-(1-(*o*-Tolyl)-1*H*-1,2,3-triazole-4-yl)cyclohexan-1-ol (**1k**):

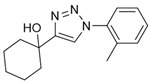



Triazole **1k** was isolated as an orange solid (yield 89%, purity: 99.6%). mp: 201–203 °C; FT-IR υ (cm^−1^): 3227, 3106, 3062, 2933, 1609, 1593, 1490; **^1^H NMR** (400 MHz, DMSO-d6) δ 8.56 (s, 1H), 7.78–7.65 (m, 2H), 7.46 (t, *J* = 7.8 Hz, 1H), 7.28 (d, *J* = 7.6 Hz, 1H), 5.00 (s, 1H), 1.96 (t, *J* = 9.9 Hz, 2H), 1.80 (s, 2H), 1.77–1.63 (m, 3H), 1.59–1.24 (m, 4H); **^13^C NMR** (101 MHz, DMSO) δ 157.10, 140.05, 137.25, 130.10, 129.38, 120.73, 119.70, 117.36, 68.42, 38.13, 25.70, 22.18, 21.38; HRMS (ESI): *m*/*z* [M+Na] calcd. for C_15_H_19_N_3_NaO: 280.142724, found: 280.142724.

Characterization of (1-(2-Chlorophenyl)-1*H*-1,2,3-triazole-4-yl)methyl benzoate (**1l**) [28]:

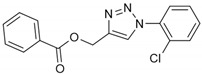



Triazole **1l** was isolated as a brown oil (yield 75%, purity: 92.8%). FT-IR υ (cm^−1^): 3227, 3067, 2958, 2133, 2106, 1714, 1601, 1584, 1494, 1451; **^1^H NMR** (400 MHz, DMSO-d6) δ 8.73 (s, 1H), 8.01 (dd, *J* = 7.0, 1.5 Hz, 2H), 7.82–7.51 (m, 8H), 5.53 (s, 2H); **^13^C NMR** (101 MHz, DMSO) δ 165.91, 142.53, 134.84, 134.04, 132.23, 131.02, 129.76, 129.56, 129.24, 129.01, 128.94, 128.92, 127.43, 58.21; HRMS (ESI): *m*/*z* [M+Na] calcd. for C_16_H_12_ClN_3_NaO_2_: 336.051025, found: 336.050532.

Characterization of (1-(*o*-Tolyl)-1*H*-1,2,3-triazole-4-yl)methyl benzoate (**1m**) [28]:

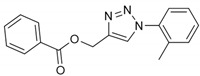



Triazole **1m** was isolated as a yellow solid (yield 95%, purity: 98.2%). mp: 150–152 °C; FT-IR υ (cm^−1^): 3147 3067, 2960, 2135, 2106, 1714, 1601, 1584; **^1^H NMR** (400 MHz, DMSO-d6) δ 8.64 (s, 1H), 8.05–7.95 (m, 2H), 7.68 (t, *J* = 7.4 Hz, 1H), 7.59–7.37 (m, 6H), 5.52 (s, 2H), 2.17 (s, 3H); **^13^C NMR** (101 MHz, DMSO) δ 165.94, 142.50, 136.59, 134.03, 133.53, 131.84, 130.36, 129.80, 129.77, 129.29, 127.47, 126.81, 126.51, 58.34, 17.87; HRMS (ESI): *m*/*z* [M+Na] calcd. for C_17_H_15_N_3_NaO_2_: 316.105647, found: 316.106402.

Characterization of (1-(4-Methoxyphenyl)-1*H*-1,2,3-triazole-4-yl)methanol (**1n**) [31,32]:

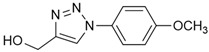



Triazole **1n** was isolated as an orange solid (yield 80%, purity 94,7%). mp: 127–129 °C. FT-IR υ (cm^−1^): 3173, 3116, 3073, 3000, 2929, 2833, 1607, 1590, 1517; **^1^H NMR** (400 MHz, DMSO-d6) δ 8.56 (s, 1H), 7.84–7.75 (m, 2H), 7.18–7.09 (m, 2H), 5.33 (t, *J* = 5.6 Hz, 1H), 4.60 (d, *J* = 5.5 Hz, 2H), 3.83 (s, 3H); **^13^C NMR** (101 MHz, DMSO) δ 149.33, 130.66, 126.36, 122.07, 121.43, 115.32, 115.09, 56.01, 55.43; HRMS (ESI): *m*/*z* [M+Na] calcd. for C_10_H_11_N_3_NaO_2_: 228.074347, found: 228.074826.

### 2.3. In Silico Prediction

DataWarrior software version 5.5.0 [33] was used to predict physicochemical properties. SwissADME service (https:www.swissadme.ch accessed on 31 may 2023) was used to predict drug oral bioavailability using radar graphics.

### 2.4. Two- and Three-Dimensional Cell Cultures

VERO cells (Rio de Janeiro Cell Bank code 0245) were cultivated in RPMI 1640 medium supplemented with 10% fetal bovine serum (FBS) at 37 °C in a humidified atmosphere of 5% CO_2_. Confluent monolayers were dissociated with trypsin-EDTA solution (0.025%), and isolated cells were seeded on culture plates or flasks, depending on the experimental assay. The VERO cell cultures were used in cytotoxicity and phenotypic screening assays and for obtaining culture-derived trypomastigotes.

Primary heart muscle cell cultures were obtained from fetuses of female Swiss Webster mice, as previously described [34]. After heart removal and ventricle fragmentation, tissue fragments were dissociated in a trypsin and collagenase type II (Worthington Biochemical Corporation, Lakewood, USA) dissociation solution. For two-dimensional (2D) cultures, the isolated cardiac cells were seeded at a density of 5 × 10^4^ cells/well in gelatin (0.1%)-coated 96-well white culture plates. Three-dimensional (3D) cardiac spheroids were obtained after seeding isolated heart muscle cells at a density of 2.5 × 10^4^ cells/well in an agarose (1%)-coated 96-well U-bottom plate, as described in [35]. Heart muscle cells cultures were cultivated in Dulbecco’s modified Eagle medium (DMEM) supplemented with 7% FBS, 2.5 mM CaCl_2_, 2% embryo extract, and 1 mM *L*-glutamin and maintained at 37 °C in a humidified atmosphere of 5% CO_2_. All procedures with animals were approved by the Animal Care and Use Committee at the Oswaldo Cruz Institute (license L-017/2022).

### 2.5. Parasites

A drug screen was carried out with *Trypanosoma cruzi* clone Dm28c genetically modified to express luciferase (Dm28c-luc), kindly provided by Dr. Cristina Henriques [36]. The genetically modified *T. cruzi* clone Dm28c (Dm28c-Luc) has the firefly luciferase gene integrated into the genome, stably expressing the luminescent enzyme. The bioluminescent signal, proportional to the number of parasites, is produced by adding a D-luciferin substrate [37]. Trypomastigotes were harvested from supernatants of VERO cell cultures, infected at a ratio of 10:1 parasite/host cells, 4 days post-infection (4 dpi). Parasites were used for phenotypic drug screening procedures. Luminescent parasites are a reliable and sensitive tool that allows precise quantification of parasite load. The use of genetically modified organisms was approved under license CQB 105/99.

### 2.6. Cytotoxicity Analysis

The toxic effect of the triazole series was evaluated on VERO cell monolayers. After seeding for 24 hours, VERO cells were treated for 72 h at 37 °C with the 1,2,3-triazole derivatives and Bz, the reference drug, with a range of concentration from 15.62 to 500 µM. Controls were performed with a non-toxic concentration of dimethyl sulfoxide (DMSO; ≤0.1%) used in the experimental assays. Cell viability was determined by measuring ATP level using a CellTiter Glo kit [38]. The luminescence read was performed using a Glomax microplate reader (Promega Corporation, Madison, WI, EUA). The cytotoxicity concentration 50 (CC_50_), the drug concentration that reduces cell viability by 50%, was calculated using linear regression. The cardiotoxic effect of the most effective compounds was also evaluated using 2D and 3D cultures. Cultures were exposed for 72 h to compound concentrations up to 500 µM, following the protocol mentioned above. At least 3 independent assays were performed in duplicate.

### 2.7. Trypanocidal Activity

All triazole analogs were analyzed for their biological activity against trypomastigote and intracellular amastigote forms of *T. cruzi* (Dm28c-Luc). Trypomastigotes (1.0 × 10^6^ parasites/well) were incubated for 24 h at 37 °C with triazole derivatives and Bz at concentrations ranging from 0.04 to 100 µM. Luciferin (300 µg/mL), a luciferase substrate, was added to parasite suspensions to evaluate trypomastigotes viability [38]. The luminescent signal was measured using a Glomax microplate reader. A maximal DMSO concentration (0.1%) was used as the negative control. The inhibitory concentration 50 (IC_50_) and 90 (IC_90_) against *T. cruzi*, which reduces the number of parasites by 50% and 90%, respectively, was calculated using linear regression. The selectivity index (SI), a ratio that measures the window between toxicity in mammalian cells and anti-*T. cruzi* activity (SI = CC_50_/IC_50_), was also determined.

Activity against intracellular amastigotes was determined in cultures of VERO cells infected by *T. cruzi* (24 h), as previously described [38]. Briefly, infected cultures were treated for 72 h at 37 °C with different concentrations of triazole derivatives (0.04–100 µM). Then, the culture supernatant was removed, and the viability of the intracellular parasites was evaluated after adding luciferin (300 µg/mL) to the cell monolayer followed by reading using the microplate reader. Anti-*T. cruzi* activity (IC_50_ and IC_90_) and SI values were determined. All experimental assays were performed at least 3 times in duplicate.

### 2.8. Drug Efficacy in 3D Microtissue

A three-dimensional cardiac model was applied as a cell culture platform to test the efficacy of promising candidates. Cardiac spheroids, with 5–7 days of culture, were infected for 24 h with *T. cruzi* Dm28c-Luc (5 × 10^5^ parasites/well) and then, after washing, incubated for 72 h at 37 °C with 15 to 30 times the IC_90_ value of the most active compounds (IC_50_ ≤ 10 µM). Microtissues were also incubated with DMSO (0.1%) and Bz (100 µM) as negative and positive controls, respectively. After luciferin addition (300 µg/mL), the luminescence was measured, and the data were expressed as arbitrary luminescence units (ALU).

### 2.9. Washout Assay

A washout assay was performed to evaluate the capacity of the promising candidates to eliminate all parasites. Thus, VERO cells, seeded in 96-well plates at a density of 1.5 × 10^4^ cells/well, were infected with *T. cruzi* (Dm28c-Luc) at a 10:1 parasite–host cell ratio (24 h). Two treatment schedules were performed: 3 days with 30, 50, and 100 µM concentrations and 10 days with 30 and 60 times the IC_50_ concentrations. After treatment, the cultures were washed with PBS and kept for the same period in RPMI 1640 medium supplemented with 10% FBS without compound pressure. The culture medium was changed every 3 or 4 days during the time course, and the supernatant was harvested for luminescence reading after luciferin (300 µg/mL) addition. At the endpoint, the monolayers were also evaluated for parasite load. The luminescent signal was read using a Glomax reader. Bz (100 µM) and DMSO (≤1%) were used as positive and negative controls, respectively.

### 2.10. Drug Combination Assay

The combination of promising candidates and Bz was performed using an isobologram method [39]. Initial concentrations were determined by ensuring that IC_50_ concentrations of monotherapy compounds remained close to half in serial dilutions (1:3–6 concentrations). Solutions at the initial concentration of each compound were prepared and then mixed in proportions of 5:0, 4:1, 3:2, 2:3, 1:4, and 0:5 (*v*/*v*) of promising compounds and Bz, respectively. The anti-*T. cruzi* activity for the serial dilutions (1:3) of the ratios was then analyzed, as previously described [39]. The IC_50_ values for each combination were used to determine the fractional inhibitory concentration index (FICI); thus, FICI(A) = IC_50_(A) in combination/IC_50_(A) alone and FICI(B) = IC_50_(B) in combination/IC_50_(B) alone. In addition, the sum of the FICIs for each proportion (∑FICI = FICI(A) + FICI(B)) and the average of the sums for the FICIs (xΣFICI) were calculated. The FICI values were plotted on an isobologram graph. The xΣFICI was applied as a classification criterion for interactions: as synergistic xΣFICI ≤ 0.5, additive xΣFICI > 0.5–1, without interaction xΣFICI > 1–4, and antagonistic for xΣFICI > 4.

### 2.11. Statistical Analysis

Data are presented as the mean and standard deviation (SD) of at least three independent experiments. All statistical analyses were performed using GraphPad Prism version 8.2 (GraphPad software, Inc., La Jolla, CA, USA). A statistical difference, calculated using an ANOVA (Kruskal–Wallis), was considered as a *p*-value ≤ 0.05.

## 3. Results and Discussion

### 3.1. In Silico Characterization of 1,2,3-Triazole Derivatives

**Figure 1:**
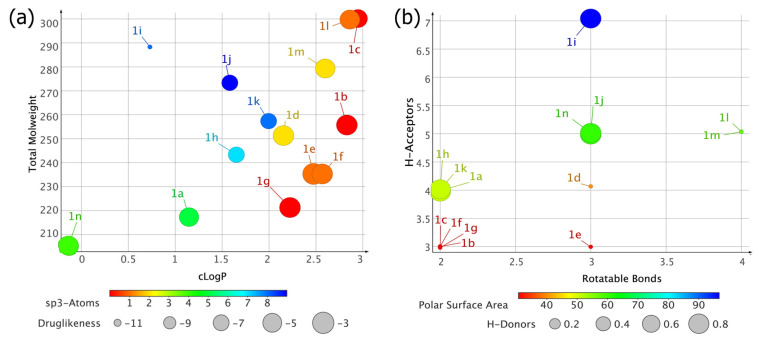
Physicochemical properties of 1,2,3-triazole derivatives. (**a**) The graphical distribution and the relationship between the compounds, according to the molecular weight (MW), lipophilicity (cLogP), fraction of sp^3^ carbon atoms (Fsp^3^), and drug-likeness. (**b**) Relationship between topological polar surface area (tPSA), rotatable bonds, number of hydrogen bond donors (H-donors), and hydrogen bond acceptors (H-Acceptors).

Computer-aided drug discovery tools have been an attractive strategy for identifying novel hits and optimizing hit-to-lead compounds [40]. *In silico* analysis has an impact on accelerating the discovery of new drugs by discarding compounds with poor physicochemical and pharmacokinetic properties in the early stages of development, thus reducing the risks of failure [41,42]. Considering this issue, the physicochemical properties of the 1,2,3-triazole derivatives were assessed with the aim of predicting the drug-likeness profile of the designed compounds. A total of 14 derivatives (**1a**–**n**) were analyzed for their compliance with Lipinski’s rule, which considers that a molecule is orally bioavailable when it has a molecular weight (MW) < 500, octanol/water partition coefficient (cLogP) < 5, number of hydrogen bond donors (HBD) < 5, and hydrogen bond acceptors (HBA) < 10. The compounds had a low MW, varying from 205.21 to 300.15 g/mol (Figure 1). The optimal cLogP range (1.520132.9) was mostly observed in 1,2,3-triazole derivatives, favoring permeation of biological barriers. Only **1i** (cLogP = 0.73) and **1n** (cLogP = −0.13) had greater polarity (Figure 1), which may favor aqueous solubility but tended to limit membrane permeability. Lipophilicity is an important property that impacts drug absorption, distribution, metabolism, excretion, and toxicity (ADMET) [43]. However, oral bioavailability is also influenced by tPSA and flexibility [44]. Our *in silico* analysis showed that all derivatives have a topological polar surface area (tPSA) < 140 Å^2^, HBD < 5, HBA < 10, and rotatable bonds (RB) < 10, with a prediction of good oral bioavailability (Figure 1). These findings were also confirmed using bioavailability radar (Supplementary Figure S2), with most of the physicochemical parameters, including lipophilicity (LIPO), size (SIZE), polarity (POLAR), solubility (INSOLU), flexibility (FLEX), and saturation (INSATU), within the physicochemical space that represents the prediction of oral bioavailability (radar pink area).

### 3.2. Cytotoxicity and Biological Activity

All triazole analogs showed good drug-likeness prediction and advanced to phenotypic screening assays against *T. cruzi* (Dm28c-Luc). Toxicity is undoubtedly the main cause of failure for drugs in clinical trials. Thus, the cytotoxicity was explored in vitro on VERO cell monolayers, with viability measured using the ATP level. Low toxicity was observed for all compounds analyzed (CC_50_ > 236 µM) except **1f** (CC_50_ = 86.8 ± 2.73 µM), which demonstrated moderate cytotoxicity (Table 1). Next, we assessed the antiparasitic effect of the 1,2,3-triazole derivatives (**1a**–**n**) (Table 1). Most compounds had low activity against *T. cruzi*, with IC_50_ > 70 µM for both trypomastigotes and intracellular amastigotes forms (Table 1). However, three derivatives, including **1d** (pIC50 = 6.67), **1f** (pIC50 = 5.91), and **1g** (pIC50 = 5.64), showed a remarkable effect against trypomastigotes, with higher potency when compared to the reference drug (Bz; pIC50 = 4.64). The effectiveness of **1d** (IC_50_ = 0.21 ± 0.03 µM), **1f** (IC_50_ = 1.23 ± 0.24 µM), and **1g** (IC_50_ = 2.28 ± 0.34 µM), with IC_50_ values 10- to 100-fold lower than Bz (IC_50_ = 22.79 ± 4.12 µM), highlights their potent activity. Compared with **1g**, the introduction of the substituents methoxy (**1d**), an electron donating group, using resonance, and methyl (**1f**), an electron donor group, using induction, in the *para* and *ortho* positions of the phenyl ring increased the activity by 11 and 1.8 times, respectively. Interestingly, **1d** (IC_50_ = 3.27 ± 0.90 µM), **1f** (IC_50_ = 3.50 ± 0.39 µM), and **1g** (IC_50_ = 6.20 ± 1.06 µM) were also the most effective compounds against intracellular amastigotes, with anti-*T. cruzi* activity comparable to Bz (IC_50_ = 4.67 ± 0.22 µM) (Table 1). Among promising candidates, **1d** (IC_90_ = 2.90 ± 0.16 µM) and **1f** (IC_90_ = 3.68 ± 0.60 µM) stood out with IC_90_ values lower than Bz (IC_90_ > 100 µM) for both trypomastigotes. However, **1d** had the highest selectivity index (SI) for trypomastigotes (SI > 2380) and intracellular amastigotes (SI > 152), suggesting a potential wide therapeutic window of this compound (Table 1).

Database analysis highlights triazole-containing heterocycles as privileged scaffolds in anti-*T. cruzi* drug development [45]. Derivatives of the 5-amino-1,2,3-triazole-4-carboxamides series have demonstrated potent in vitro biological activity [46]. The most active compound in this series (analog 58) was analyzed in a mouse model of acute and chronic infection using *T. cruzi* clone CL-luciferase. Oral treatment (50 mg/kg for 20 days) reduced parasite burden but was unable to induce sterile cure, with relapse after mice immunosuppression [46]. The new 1,2,3-triazole-2-nitroimidazole series also revealed high potency against *T. cruzi in vitro*, with 5-fold more activity than Bz [23]. Recently, new 1,2,3-triazole-selenide hybrids also showed anti-*T. cruzi* activity comparable to the reference drug [47]. Our data highlight the potential of 1-(4-diphenyl)-1*H*-1,2,3-triazole derivatives against *T. cruzi*, with a nanomolar activity for trypomastigotes (100-fold more active than Bz) and similar potency to Bz for intracellular parasites. Triazole-based molecules have been identified as potent inhibitors of *T. cruzi* drug targets [48,49,50,51]. Two new 1,2,3-triazole derivatives (**9** and **10**), synthesized from natural phenylpropanoids, interact with the active site of cruzain with molecular docking and reduce parasitemia in *T. cruzi* experimental infection in vivo [48]. Bioactive derivatives consisting of 1,2,3-triazole and 1,2,4-triazole heterocycles have been emphasized as inhibitors of enzymes involved in *T. cruzi* metabolism, including trans-sialidase [49], trypanothione synthetase [50], and cruzain [51].

### 3.3. Cardiotoxic Effect of 1,2,3-Triazole Candidates

Drug-induced cardiotoxicity remains a major cause of attrition in drug development [52,53]. In this regard, we explored the cardiotoxic effect of promising candidates (**1d**, **1f**, and **1g**) in a 2D and 3D primary culture of heart muscle cells. Derivatives **1d** and **1g** did not induce a cardiotoxic effect, showing CC_50_ > 500 µM in both 2D and 3D culture models (Table 2). Derivative **1f** revealed a low toxicity (CC_50_ = 111.33 ± 10.06 µM) on cardiac monolayers (2D) but no cardiotoxic effect was detected on 3D cardiac microtissue (CC_50_ > 500 µM). In general, 2D cultures are more susceptible to compound-induced toxicity than 3D microtissues [54]. The difference in drug response may be related to physical and physiological properties, such as morphology, distribution of surface receptors, proliferative stage, and pH level, promoting drug susceptibility or resistance [54].

### 3.4. Drug Efficacy in T. cruzi-Infected 3D Cardiac Spheroid

**Figure 2:**
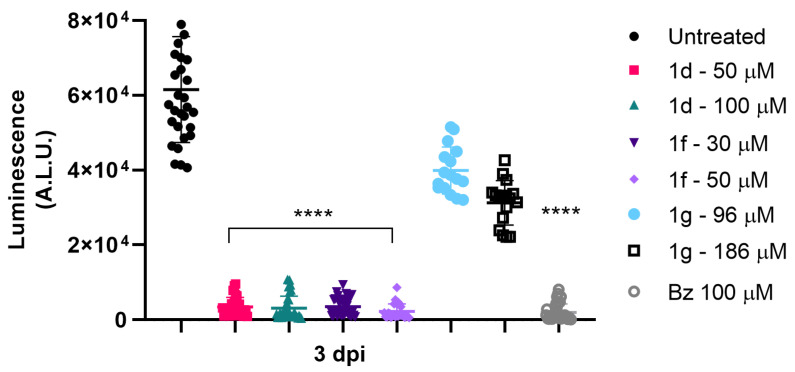
Biological activity of 1,2,3-triazole derivatives (**1d**, **1f** and **1g**) in *T. cruzi*-infected 3D cardiac spheroids. Anti-*T. cruzi* activity of the compounds was determined using quantification of the luminescent signal, measured in arbitrary luminescence units (A.L.Us.) Note that **1d** and **1f** in both concentrations significantly inhibited the viability of the parasites, exhibiting efficacy comparable to Bz. A one-way ANOVA test was used to determine the statistical significance relative to the untreated and treated groups, *p* ≤ 0.0001 (****).

Bridging the gap between in vitro and animal models in drug discovery, 3D cardiac microtissue, which has a more realistic physiological microenvironment of in vivo tissues compared to 2D cultures [55], was also applied to evaluate the efficacy of the compounds. Organoid cultures have been highlighted as a drug screening platform to improve the efficiency of drug development [56], providing more robust data to proceed with in vivo preclinical studies. Herein, *T. cruzi*-infected cardiac spheroids were used to address the effectiveness of promising candidates since the cardiac cells are the main target of infection by *T. cruzi*. The 3D microtissues were treated with the promising candidates at concentrations of 15 to 30 times the IC_50_, except for **1f**, whose maximum concentration reached 50 µM. Derivatives **1d** and **1f** were able to significantly reduce the parasite load in 3D cardiac microtissue, showing an effective diffusion and effectiveness of the 1,2,3-triazole candidates (Figure 2). These derivatives showed an anti-*T. cruzi* effect similar to Bz, even at low concentrations (≤50 µM). In contrast, **1g** did not effectively decrease the total number of viable intracellular parasites (Figure 2).

Our data reinforce using 3D primary cardiac microtissue as a suitable model to assess drug efficacy, which can improve the translation potential for anti-*T. cruzi* drugs, reducing gaps between in vitro and in vivo models. Efficient preclinical screening is essential to avoid therapeutic failures in clinical development and the use of 3D culture models has been widely encouraged to accurately screen candidate drugs [57]. The 3D culture model has attracted attention in the development of new drugs and vaccines for infectious diseases [58]. This tool has allowed advances in the discovery of antimicrobial [59,60] and antiparasitic drugs, but it has been rarely used in Chagas disease drug screening. H9c2 cardiomyoblast 3D spheroids were recently used to evaluate the anti-*T. cruzi* effect of nucleoside analogs, showing potent activity against intracellular amastigotes [61]. HepG2 monolayers and 3D cultures were also exploited to investigate the hepatotoxicity of atorvastatin–aminoquinoline derivatives screened against *T. cruzi* [62]. The efficacy of pyrazole derivatives, pyrazole–imidazoline and pyrazole–thiazoline scaffolds, against *T. cruzi* was also explored using VERO cell spheroids [38,39]. In cancer research, 3D microtissue has been widely used as a powerful tool for anti-cancer drug screening since its complex organization represents the tumor microenvironment with greater reliability, interfering with drug diffusion and efficacy [63,64]. Therefore, the introduction of 3D culture models in preclinical drug screening platforms may overcome the drawbacks of immortalized cell lineage (2D culture) and have the potential to reduce translational lacunas between in vitro and animal models.

### 3.5. Drug Potential to Inhibit Parasite Resurgence

**Figure 3:**
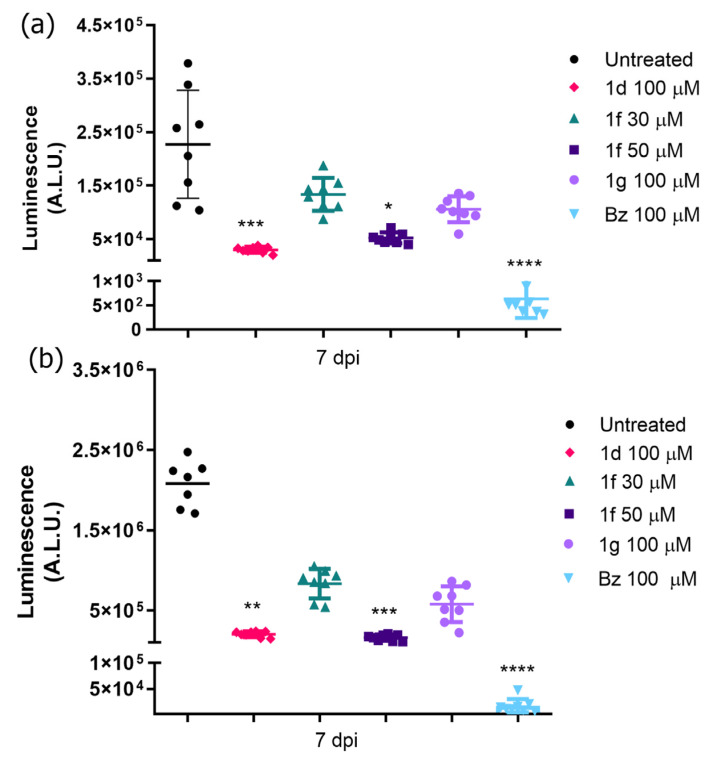
Short-term washout assay with 1,2,3-triazole-treated *T. cruzi*-infected VERO cells. Treatment (3 days) of Dm28c-luc-infected VERO cells with **1d**, **1f**, and **1g** at different concentrations (30, 50, or 100 µM) followed by 3 days in the absence of treatment. Detection of trypomastigotes in the culture supernatant (**a**) and intracellular parasites in VERO cell monolayers (**b**) was revealed using arbitrary luminescence units (A.L.Us.). Statistical significance, in relation to the untreated group, was determined using a one-way ANOVA test, with *p* ≤ 0.0001 (****), *p* ≤ 0.001 (***), *p* ≤ 0.01 (**) and *p* ≤ 0.05 (*).

An important open question was whether treatment with the promising candidates could induce a sterile cure in vitro. To assess drug efficacy more accurately, monolayers of VERO cells infected with *T. cruzi* (24 h) were exposed to short-term treatment (3 days) followed by cultivation for 3 days without compound pressure. Both the release of trypomastigotes in the culture supernatant and the presence of intracellular amastigotes in cell monolayers were determined after reading the luminescent signal (arbitrary luminescence unit; A.L.U.). Our results demonstrated significant inhibition of released trypomastigotes and cell monolayer infection. Among the promising candidates analyzed, **1d** (100 µM) and **1f** (50 µM) were the most effective compounds (Figure 3). In contrast, **1f** (30 µM) and **1g** (100 µM) did not significantly reduce the parasite load. Despite the potent activity of **1d** (100 µM) and **1f** (50 µM), achieving an approximately 10- to 12-fold reduction, respectively, in parasite load compared to untreated cultures, these derivatives did not prevent parasite resurgence (Figure 3). Treatment with Bz (100 µM) potentially inhibited the infection progression, with a few parasites released and low infection of the cell monolayer (Figure 3), indicating failure to eliminate parasites with short-term treatment.

**Figure 4:**
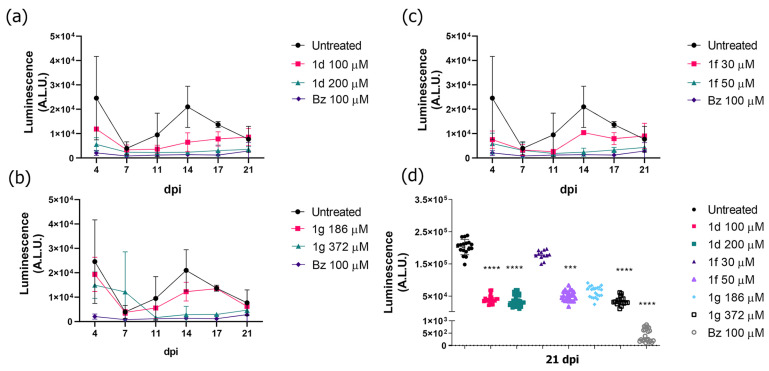
Long-term (21 days) washout assay with 1,2,3-triazole-treated, *T. cruzi*-infected VERO cells. Infected monolayers (24 h) were treated for 10 days with promising candidates (**1d**, **1f,** and **1g)** followed by another 10 days in the absence of treatment pressure. Detection of trypomastigotes released in the culture supernatant after treatment with **1d** (**a**), **1f** (**b**), and **1g** (**c**) at different concentrations (30 or 60 times IC_50_). (**d**) Viable intracellular parasites in the cell monolayer were revealed using arbitrary luminescence units. Statistical significance, in relation to the untreated group, was determined using a one-way ANOVA test, with *p* ≤ 0.0001 (****) and *p* ≤ 0.001 (***).

The promising results showing that the analogs potentially decreased infection without drug pressure led us to investigate their ability to induce sterile cure using a long-term treatment assay. Thus, *T. cruzi*-infected VERO cell monolayers were treated for 10 days with the promising candidates, and the reversibility was monitored for another 10 days. The maximum concentration of **1d** and **1f** showed a similar inhibitory effect to Bz on the release of trypomastigotes (Figure 4). Although the 1,2,3-triazole derivatives potentially reduced the parasite load, none inhibited infection reactivation (Figure 4). However, prolonged treatment with Bz also failed to induce a sterile cure (Figure 4). Treatment of VERO cells infected with Silvio X10/7 with Bz (12.5 to 50 times EC_50_) for 8 days also did not prevent relapse, but a lack of recrudescence was evidenced for 60 days after 16 days of treatment with 25 to 50 times EC_50_ [65]. Interestingly, treatment for 8 days with Bz was able to delay the resurgence of the parasite, even using a highly proliferative strain (Silvio X10/7). This fact was not observed in our analysis, where a very low, but continuous, release of trypomastigotes was evidenced. It is possible that this effect is related to the maximum concentration of Bz used (20 times the IC_50_) or the susceptibility of *T. cruzi*.

**Figure 5:**
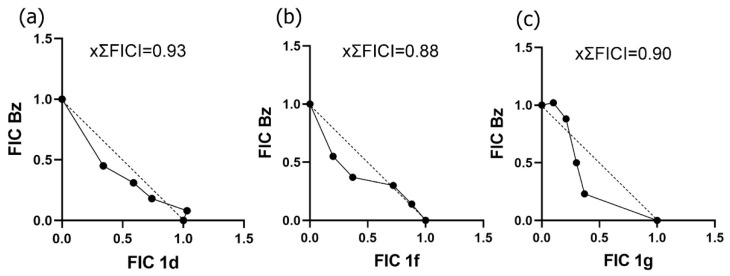
in vitro combinatorial activity of the promising 1,2,3-triazole derivatives and Bz against amastigotes of *T. cruzi.* The isobologram graph was constructed by plotting the FICI values for each proportion of compounds **1d** (**a**), **1f** (**b**), and **1g** (**c**) in combination with Bz (Solid line). Diagonal drawn dashed line represents the expected FICI points of combinations given dose-additivity.

Drug combinations with distinct pharmacological compounds have been applied as a strategy to improve anti-*T. cruzi* activity. The resulting in vitro effect of Bz combined with the promising candidates against intracellular amastigote was analyzed with a luminescent assay, using *T. cruzi* Dm28c-Luc. Drug pairs, combined at six different ratios (0:5, 1:4, 2:3, 3:2, 4:1, and 5:0), allowed the calculation of FICI, ΣFICI, and xΣFIC. The data revealed an additive effect of the 1,2,3-triazole derivatives **1d** (xΣFICI = 0.93), **1f** (xΣFICI = 0.88), and **1g** (xΣFICI = 0.90) with Bz (Figure 5), indicating that the combination effect is equal to the sum of their activity alone.

## 4. Conclusions

This study integrated virtual analysis and phenotypic drug screening to predict the oral bioavailability and evaluate the trypanocidal effect of 1,2,3-triazole derivatives, respectively. Three-dimensional spheroids and reversibility assay are suitable in vitro preclinical models to improve the translational success of drug candidates. The reported data highlight the activity of **1d**, **1f,** and **1g** against *T. cruzi*, showing good permeability and efficacy in 3D cardiac microtissue. However, the washout assay demonstrated that while the analogs potentially reduced the parasite load, they did not prevent parasite resurgence. The combination of Bz and triazole promising candidates induced an additive effect with the isobologram analysis, suggesting that combination treatment may lead to a positive outcome in vivo. New 1,2,3-triazole derivatives, containing methoxy and methyl substituents on the phenyl ring, will be designed, aiming to improve the compounds’ anti-*T. cruzi* activity.

## Figures and Tables

**Table 1:** Cytotoxicity and trypanocidal effect to 1,2,3-triazole derivatives.

	Trypanocidal Activity Mean ± SD (µM)						Cytotoxicity
Compounds	Trypomastigotes			Intracellular Amastigotes			VERO Cells
	**IC_50_**	**IC_90_**	**SI**	**IC_50_**	**IC_90_**	**SI**	**CC50**
1a	>100	>100	Nd	>100	Nd	Nd	>500
1b	>100	>100	Nd	>100	Nd	Nd	>500
1c	>100	>100	Nd	>100	Nd	Nd	>500
1d	0.21 ± 0.03	2.90 ± 0.16	>2380	3.27 ± 0.90	>100	>152	>500
1e	>100	>100	Nd	83.24 ± 2.27	Nd	>6	>500
1f	1.23 ± 0.24	3.68 ± 0.60	70.5	3.50 ± 0.39	30.42 ± 0.4	24.8	86.8 ± 2.73
1g	2.28 ± 0.34	>100	>219	6.20 ± 1.06	>100	>80.6	>500
1h	>100	Nd	Nd	>100	Nd	Nd	>500
1i	>100	Nd	Nd	>100	Nd	Nd	>500
1j	>100	Nd	Nd	>100	Nd	Nd	>500
1k	>100	Nd	Nd	>100	Nd	Nd	>500
1l	80.42 ± 3.56	>100	2.94	77.90 ± 2.00	>100	3.03	236.41 ± 17.87
1m	>100	Nd	Nd	>100	Nd	Nd	>500
1n	>100	Nd	Nd	>100	Nd	Nd	>500
Bz	22.79 ± 4.12	>100	>100	4.67 ± 0.22	21.69 ± 2.37	>107	>500

Mean values for IC_50_ and IC_90_ from three independent experiments ± standard deviation (SD); IC_50_: concentration that inhibits parasite viability by 50%; CC_50_: concentration that reduces the viability of VERO cells by 50%; Nd = Not determined; Selectivity index (SI) = CC_50_ of VERO cells/IC_50_ of trypomastigotes and intracellular amastigotes forms of *T. cruzi*.

**Table 2:** Cardiotoxic effect of 1,2,3-triazole derivatives.

Compounds	Toxicity (Mean ± SD µM)	
	2D Culture	3D Culture
1d	>500	>500
1f	111.33 ± 10.06	>500
1g	>500	>500

Mean value for CC_50_ calculated using three independent experiments ± standard deviation (SD). CC_50_: concentration that reduces heart muscle cell viability by 50%.

## Data Availability

Not applicable.

## Supplementary Materials

The following supporting information can be downloaded at: https://www.mdpi.com/article/10.3390/biology12091222/s1, Figure S1: NMR spectra of the compounds; Figure S2: Oral bioavailability radar analysis.
