# Supplementary material for: Antitrypanosomal Activity of 1,2,3-Triazole-Based Hybrids Evaluated Using In Vitro Preclinical Translational Models
Source: Biology (Basel). 2023 Sep 8;12(9):1222. doi: 10.3390/biology12091222 (PMC10525445; doi:10.3390/biology12091222)
Supplement: Supplementary file 1 [file biology-12-01222-s001.zip › biology-2467495-supplementary.pdf]

Supporting Information

Triazole Synthesis

General Procedures for CuAAC of Organic Azides with Alkynes to 1,4-Disubstituted 1,2,3-Triazoles (6a-r).

The synthesis of all 1,4-disubstituted 1,2,3-triazoles 1(a - r) was performed using the adjusted methodology of Shao et al. (1). In scheme 1, 1.0 mmol of arylazide (4a-i), 1.1 mmol of alkyne (5a-d), and 5 mL of H_2O were stirred with 50 µL of glacial acetic acid (HAC) for 60 s. Then, 1 mg of Cu_2O (0.7 mol%) was added to the mixture, which was stirred at room temperature for 30-60 min. Subsequently, the mixture was extracted with ethyl acetate (3 x 15 ml), and the combined organic phases were dried with Na_2SO_4 and concentrated on a rotary evaporator to obtain the corresponding 1,4-disubstituted 1,2,3-triazole. The triazoles 1d, 1h, 1i, 1j, and 1k were crystallized from acetonitrile:hexane (1:4).

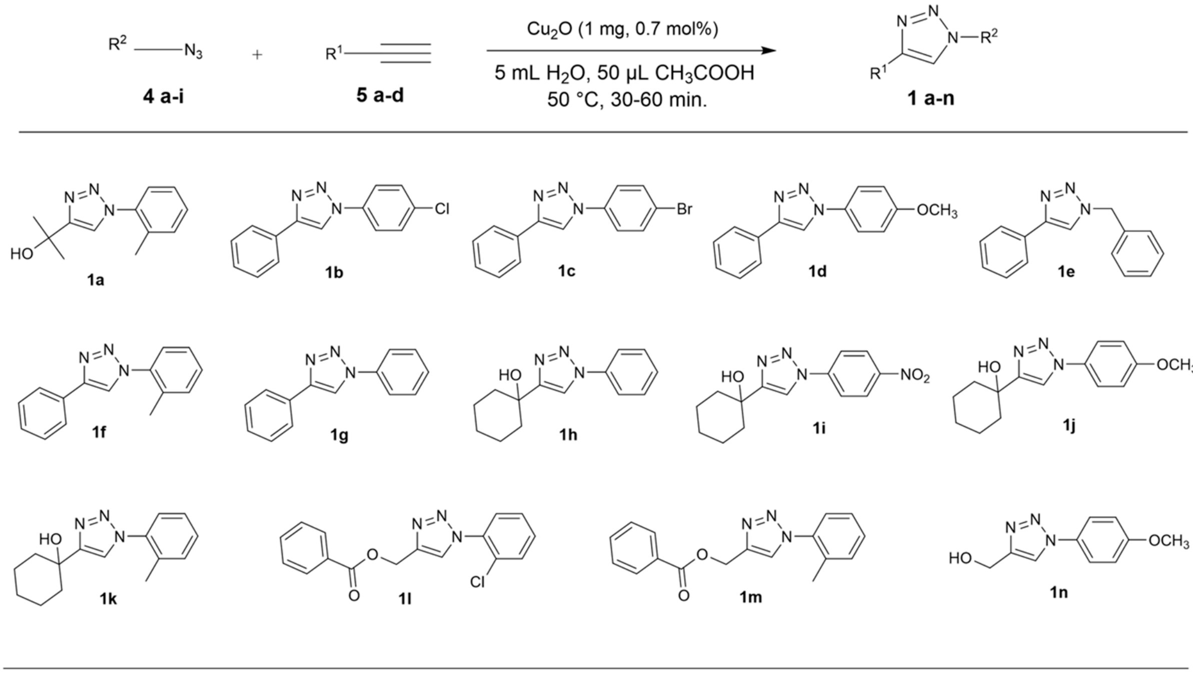

Scheme 1. Condition of the CuAAC reaction between aryl azides (4a-i) and alkynes (5a-d) to obtain 1,4-disubstituted 1,2,3-triazole (1a-r).

8. Characterization data for triazoles

Characterization of 2-(1-(o-tolyl)-1H-1,2,3-triazol-4-yl)propan-2-ol (1a)(2)**:**

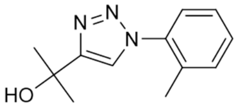

Triazole **1a** was isolated as a pale brown solid (yield 90 %, purity: 99.4%). mp: 120-122 ºC; FT-IR υ (cm^{-1}): 3336, 3120, 2974, 2927, 1582, 1498; **^{1}H NMR** (400 MHz, DMSO-d6) δ 8.20 (s, 1H), 7.47 (dd, J = 4.4, 2.0 Hz, 2H), 7.40 (dd, J = 4.3, 2.0 Hz, 2H), 5.21 (s, 1H), 2.16 (s, 3H), 1.54 (s, 6H); **^{13}C NMR** (101 MHz, DMSO) δ 156.28, 136.97, 133.43, 131.78, 130.01, 127.39, 126.38, 122.60, 67.46, 31.13, 17.94; HRMS (ESI): m/z [M+Na] calcd. for $C_{12}H_{15}N_3O$: 240.110733, found: 240.110674

Characterization of 1-(4-chlorophenyl)-4-phenyl-1*H*-1,2,3-triazole (1b)(2)**:**

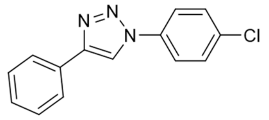

Triazole **1b** was isolated as a pale-yellow solid (yield 90 %, purity: 97.0%). mp: 218-220 ºC; FT-IR υ (cm^{-1}): 3120, 3095 3052, 1500, 1480; **^{1}H NMR** (400 MHz, DMSO-d6) δ 9.33 (s, 1H), 8.03 – 7.98 (m, 2H), 7.97 – 7.91 (m, 2H), 7.75 – 7.69 (m, 2H), 7.55 – 7.48 (m, 2H), 7.43 – 7.37 (m, 1H); **^{13}C NMR** (101 MHz, DMSO) δ 147.92, 135.90, 133.45, 130.54, 130.40, 129.51, 128.83, 125.82, 122.15, 120.18; HRMS (ESI): m/z [M+Na] calcd. for $C_{14}H_{10}ClN_3$: 278.045546, found: 278.044930.

Characterization of 1-(4-bromophenyl)-4-phenyl-1*H*-1,2,3-triazole (1c)(2)**:**

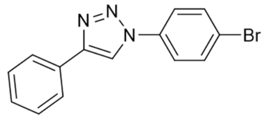

Triazole **1c** was isolated as a white solid (yield 90%, purity: 98.5%). mp: 222-225 °C; FT-IR υ (cm^{-1}): 3120, 3095 3056, 2960, 2917, 1500, 1480; **^{1}H NMR** (400 MHz, DMSO-d6) δ 9.33 (s, 1H), 7.94 (dd, J = 8.6, 1.7 Hz, 4H), 7.88 – 7.82 (m, 2H), 7.51 (dd, J = 8.4, 7.0 Hz, 2H), 7.43 – 7.37 (m, 1H); **^{13}C NMR** (101 MHz, DMSO) δ 147.94, 136.29, 133.32, 130.52, 129.51, 128.84, 125.82, 122.38, 121.82, 120.12; HRMS (ESI): m/z [M+Na] calcd. for $C_{14}H_{10}BrN_3$: 321.995030, found: 321.994100.

Characterization of 1-(4-methoxyphenyl)-4-phenyl-1*H*-1,2,3-triazole (1d)(2,3)**:**

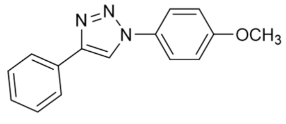

Triazole **1d** was isolated as a yellow solid (yield 92%, purity: 99.8%). mp: 159-160 °C; FT-IR υ (cm^{-1}): 3120, 3095 2960, 2935, 2837, 1607, 1515; **^{1}H NMR** 1H NMR (400 MHz, DMSO-d6) δ 9.19 (s, 1H), 7.97 – 7.92 (m, 2H), 7.89 – 7.83 (m, 2H), 7.53 – 7.47 (m, 2H), 7.38 (t, J = 7.4 Hz, 1H), 7.21 – 7.15 (m, 2H), 3.85 (s, 3H); **^{13}C NMR** (101 MHz, DMSO) δ 159.14, 146.94, 130.19, 129.89, 128.81, 127.99, 125.12, 121.52, 119.44, 114.76, 55.43; HRMS (ESI): m/z [M+Na] calcd. for $C_{15}H_{13}N_3O$: 274.095083.100168, found: 274.094521.

Characterization of 1-benzyl-4-phenyl-1H-1,2,3-triazole (1e)(4)**:**

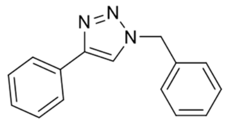

Triazole **1e** was isolated as a yellow solid (yield 95%, purity: 99.5%). mp: 123-124 °C; FT-IR υ (cm^{-1}): 3032, 2931, 2921, 2853, 1603, 1455, 1334; **^{1}H NMR** (400 MHz, DMSO-d6) δ 8.64 (s, 1H), 7.88 – 7.82 (m, 2H), 7.48 – 7.30 (m, 8H), 5.65 (s, 2H); **^{13}C NMR** 13C NMR (101 MHz, DMSO) δ 147.12, 136.47, 131.10, 129.36, 129.27, 128.64, 128.37, 128.35, 125.62, 122.03, 53.50; HRMS (ESI): m/z [M+Na] calcd. for $C_{15}H_{13}N_3$: 258.100168, found: 258.100341.

Characterization of 4-phenyl-1-(*m*-tolyl)-1*H*-1,2,3-triazole(1f):

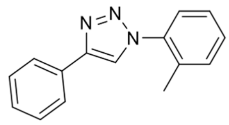

Triazole **1f** was isolated as an orange solid (yield 79%, purity: 99.7%). mp: 83-84 °C. FT-IR υ (cm^{-1}): 3122, 3095 3052, 3021, 2917, 2849, 1607, 1591; **^{1}H NMR** (400 MHz, DMSO-d6) δ 9.28 (s, 1H), 7.99 – 7.93 (m, 2H), 7.80 (s, 1H), 7.74 (s, 1H), 7.51 (td, J = 7.8, 3.0 Hz, 3H), 7.42 – 7.31 (m, 2H), 2.45 (s, 3H); **^{13}C NMR** (101 MHz, DMSO) δ 147.71, 140.16, 137.06, 130.73, 130.21, 129.78, 129.47, 128.70, 125.79, 120.87, 120.03, 117.57, 21.42; HRMS (ESI): m/z [M+Na] calcd. for $C_{15}H_{13}N_3$: 258.100168, found: 258.100587.

Characterization of 1-(4-diphenyl)-1*H*-1,2,3-triazole (1g)(2,5):

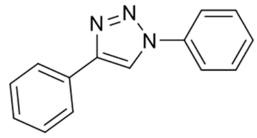

Triazole **1g** was isolated as an off white solid (yield 89%, purity: 99.5%). mp: 171-172 °C; FT-IR υ (cm^{-1}): 3119, 3084 3052, 2945, 2909, 1498, 1475; **^{1}H NMR** (400 MHz, DMSO-d6) δ 9.31 (s, 1H), 8.00 – 7.93 (m, 4H), 7.65 (dd, J = 8.6, 7.2 Hz, 2H), 7.57 – 7.48 (m, 3H), 7.43 – 7.37 (m, 1H); **^{13}C NMR** (101 MHz, DMSO) δ 147.79, 137.10, 130.70, 130.42, 129.48, 129.21, 128.73, 125.82, 120.48, 120.09; HRMS (ESI): m/z [M+Na] calcd. for $C_{14}H_{11}N_3$: 244.084518, found: 244.085003.

Characterization of 1-(1-phenyl-1H-1,2,3-triazol-4-yl)cyclohexan-1-ol (1h)(6):

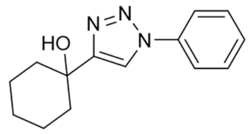

Triazole **1h** was isolated as a light white solid (yield 89%, purity: 99.8%). mp:188-190 ºC; FT-IR υ (cm^{-1}): 3223, 3101 3056, 2927, 2906, 2851, 1597, 1498; **^{1}H NMR** (400 MHz, DMSO-d_6) δ 8.62 – 8.57 (m, 1H), 7.95 – 7.87 (m, 2H), 7.59 (t, J = 7.9 Hz, 2H), 7.51 – 7.45 (m, 1H), 5.02 (s, 1H), 2.02 – 1.90 (m, 2H), 1.83 – 1.72 (m, 3H), 1.68 (d, J = 10.5 Hz, 1H), 1.47 (s, 1H), 1.38 – 1.28 (m, 1H); **^{13}C NMR** (101 MHz, DMSO) δ 157.24, 137.29, 130.32, 128.80, 120.28, 119.73, 68.43, 38.13, 25.70, 22.16. HRMS (ESI): m/z [M+Na] calcd. for $C_{14}H_{17}N_3O$: 266.126383, found: 266.127590.

Characterization of 1-(1-(4-nitrophenyl)-1H-1,2,3-triazol-4-yl)cyclohexan-1-ol (1i)(6):

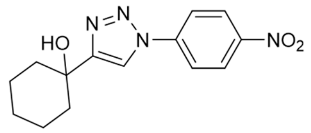

Triazole **1i** was isolated as a light white solid (yield 89%, purity 99.7%). mp: 209-210 °C; FT-IR υ (cm^{-1}): 3216, 3116, 3067, 2935, 2853, 1597, 1525, 1504; **^{1}H NMR** (400 MHz, DMSO-d_6) δ 8.84 (s, 1H), 8.48 – 8.40 (m, 2H), 8.29 – 8.22 (m, 2H), 5.11 (s, 1H), 2.03 – 1.91 (m, 2H), 1.74 (dt, J = 33.7, 11.8 Hz, 4H), 1.60 – 1.40 (m, 3H), 1.32 (q, J = 10.2 Hz, 1H); **^{13}C NMR** (101 MHz, DMSO) δ 156.79, 145.86, 140.47, 124.97, 119.69, 119.22, 67.37, 36.96, 24.59, 21.05; HRMS (ESI): m/z [M+Na] calcd. for $C_{14}H_{16}N_4O_3$: 311.111461, found: 311.111942.

Characterization of 1-(1-(4-methoxyphenyl)-1H-1,2,3-triazol-4-yl)cyclohexan-1-ol (1j):

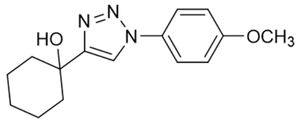

Triazole **1j** was isolated as a pale yellow solid (yield 89%, purity: 99.8%). mp: 174-175 °C; FT-IR υ (cm^{-1}): 3221, 3101, 3056, 2929, 2851, 2833, 1611, 1591, 1541, 1515; ^{1}**H NMR** (400 MHz, DMSO-*d*$_6$) δ 8.48 (s, 1H), 7.80 (d, J = 9.1 Hz, 2H), 7.12 (d, J = 9.0 Hz, 2H), 4.99 (s, 1H), 3.83 (s, 3H), 1.95 (t, J = 11.9 Hz, 2H), 1.74 (dd, J = 28.3, 15.1 Hz, 4H), 1.54 (s, 1H), 1.49 – 1.41 (m, 2H), 1.38 – 1.27 (m, 1H); ^{13}**C NMR** (101 MHz, DMSO) δ 159.46, 157.01, 130.76, 121.93, 119.69, 115.29, 68.44, 55.99, 38.15, 25.70, 22.17; HRMS (ESI): m/z [M+Na] calcd. for $C_{15}H_{19}N_3O_2$: 296.136948, found: 296.137228.

Characterization of 1-(1-(o-tolyl)-1H-1,2,3-triazol-4-yl)cyclohexan-1-ol (1k):

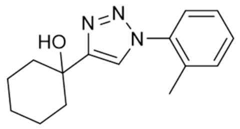

Triazole **1k** was isolated as an orange solid (yield 89%, purity: 99.8%). mp: 201-203 °C; FT-IR υ (cm^{-1}): 3227, 3106, 3062, 2933, 1609, 1593, 1490; ^{1}**H NMR** (400 MHz, DMSO-*d*$_6$) δ 8.56 (s, 1H), 7.78 – 7.65 (m, 2H), 7.46 (t, J = 7.8 Hz, 1H), 7.28 (d, J = 7.6 Hz, 1H), 5.00 (s, 1H), 1.96 (t, J = 9.9 Hz, 2H), 1.80 (s, 2H), 1.77 – 1.63 (m, 3H), 1.59 – 1.24 (m, 4H); ^{13}**C NMR** (101 MHz, DMSO) δ 157.10, 140.05, 137.25, 130.10, 129.38, 120.73, 119.70, 117.36, 68.42, 38.13, 25.70, 22.18, 21.38; HRMS (ESI): m/z [M+Na] calcd. for $C_{15}H_{19}N_3O$: 280.142724, found: 280.142724.

Characterization of (1-(2-chlorophenyl)-1H-1,2,3-triazol-4-yl)methyl benzoate (1l)(4):

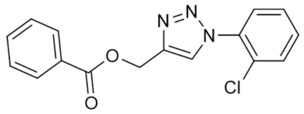

Triazole **1l** was isolated as a brown oil (yield 75%, purity: 93.2%). FT-IR υ (cm^{-1}): 3227, 3067, 2958, 2133, 2106, 1714, 1601, 1584, 1494, 1451; ^{1}**H NMR** (400 MHz, DMSO-*d*$_6$) δ 8.73 (s, 1H), 8.01 (dd, J = 7.0, 1.5 Hz, 2H), 7.82 – 7.51 (m, 8H), 5.53 (s, 2H); ^{13}**C NMR** (101 MHz, DMSO) δ 165.91, 142.53, 134.84, 134.04, 132.23, 131.02, 129.76, 129.56, 129.24, 129.01, 128.94, 128.92, 127.43, 58.21; HRMS (ESI): m/z [M+Na] calcd. for $C_{16}H_{12}ClN_3O_2$: 336.051025, found: 336.050532.

Characterization of (1-(o-tolyl)-1H-1,2,3-triazol-4-yl)methyl benzoate (1m)(4):

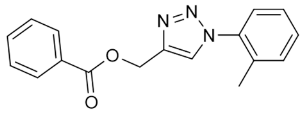

Triazole 1**m** was isolated as a yellow solid (yield 95%, purity: 98.2%). mp: 150-152 °C; FT-IR υ (cm^{-1}): 3147 3067, 2960, 2135, 2106, 1714, 1601, 1584; ^{1}**H NMR** (400 MHz, DMSO-*d*$_6$) δ 8.64 (s, 1H), 8.05 – 7.95 (m, 2H), 7.68 (t, J = 7.4 Hz, 1H), 7.59 – 7.37 (m, 6H), 5.52 (s, 2H), 2.17 (s, 3H); ^{13}**C NMR** (101 MHz, DMSO) δ 165.94, 142.50, 136.59, 134.03, 133.53, 131.84, 130.36, 129.80, 129.77, 129.29, 127.47, 126.81, 126.51, 58.34, 17.87; HRMS (ESI): m/z [M+Na] calcd. for $C_{17}H_{15}N_3O_2$: 316.105647, found: 316.106402.

Characterization of (1-(4-methoxyphenyl)-1H-1,2,3-triazol-4-yl)methanol (1n)(7,8):

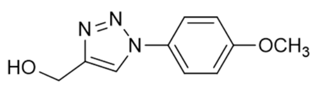

Triazole **1n** was isolated as an orange solid (yield 80%, purity 94,7%). mp: 127-129 °C; FT-IR υ (cm^{-1}): 3173, 3116, 3073, 3000, 2929, 2833, 1607, 1590, 1517; ^{1}**H NMR** (400 MHz, DMSO-d6) δ 8.56 (s, 1H), 7.84 – 7.75 (m, 2H), 7.18 – 7.09 (m, 2H), 5.33 (t, J = 5.6 Hz, 1H), 4.60 (d, J = 5.5 Hz, 2H), 3.83 (s, 3H); ^{13}**C NMR** (101 MHz, DMSO) δ 149.33, 130.66, 126.36, 122.07, 121.43, 115.32, 115.09, 56.01, 55.43. HRMS (ESI): m/z [M+Na] calcd. for $C_{10}H_{11}N_3O_2$: 228.074347, found: 228.074826.

9. References

1. Shao C, Zhu R, Luo S, Zhang Q, Wang X, Hu Y. Copper(I) oxide and benzoic acid 'on water': a highly practical and efficient catalytic system for copper(I)-catalyzed azide–alkyne cycloaddition. Tetrahedron Lett [Internet]. julho de 2011;52(29):3782–5. Available at: https://linkinghub.elsevier.com/retrieve/pii/S0040403911008082

2. Gu J, Fang Z, Yang Z, Li X, Zhu N, Wan L, et al. triazoles under metal- and azide-free conditions. 2016;1–24.

3. Meng X, Xu X, Gao T, Chen B. Zn/C-catalyzed cycloaddition of azides and aryl alkynes. European J Org Chem. 2010;(28):5409–14.

4. Alonso F, Moglie Y, Radivoy G, Yus M. Unsupported Copper Nanoparticles in the 1,3-Dipolar Cycloaddition of Terminal Alkynes and Azides. European J Org Chem [Internet]. abril de 2010;2010(10):1875–84. Available at: http://doi.wiley.com/10.1002/ejoc.201101815

5. Sarkar A, Mukherjee T, Kapoor S. PVP-stabilized copper nanoparticles: A reusable catalyst for "click" reaction between terminal alkynes and azides in nonaqueous solvents. J Phys Chem C. 2008;112(9):3334–40.

6. Bakherad M, Ghalenoei AK, Keivanloo A. Synthesis of 1,4-Disubstituted 1,2,3-Triazoles via 1,3-Dipolar Cycloaddition/C–N Coupling of Propargyl Alcohols/amines and Aryl Azides. J Heterocycl Chem [Internet]. 31 de dezembro de 2018;55(12):2683–92. Available at: https://onlinelibrary.wiley.com/doi/10.1002/jhet.3325

7. Boechat N, Ferreira VF, Ferreira SB, Ferreira M de LG, da Silva F de C, Bastos MM, et al. Novel 1,2,3-Triazole Derivatives for Use against Mycobacterium tuberculosis H37Rv (ATCC 27294) Strain. J Med Chem [Internet]. 8 de setembro de 2011;54(17):5988–99. Available at: https://pubs.acs.org/doi/10.1021/jm2003624

8. Gannarapu MR, Vasamsetti SB, Punna N, Kotamraju S, Banda N. Synthesis of novel 1-substituted triazole linked 1,2-benzothiazine 1,1-dioxido propenone derivatives as potent anti-inflammatory agents and inhibitors of monocyte-to-macrophage differentiation. Medchemcomm [Internet]. 2015;6(8):1494–500. Available at: http://xlink.rsc.org/?DOI=C5MD00171D
